# Eradicating mass spectrometric glycan rearrangement by utilizing free radicals†
†Electronic supplementary information (ESI) available. See DOI: 10.1039/c6sc01371f


**DOI:** 10.1039/c6sc01371f

**Published:** 2016-05-05

**Authors:** Nikunj Desai, Daniel A. Thomas, Jungeun Lee, Jinshan Gao, J. L. Beauchamp

**Affiliations:** a Department of Chemistry and Biochemistry , Center for Quantitative Obesity Research , Montclair State University , 1 Normal Avenue , Montclair , NJ 07043 , USA . Email: gaoj@montclair.edu ; Fax: +1-973-655-7772; b Arthur Amos Noyes Laboratory of Chemical Physics , California Institute of Technology , 1200 East California Blvd , Pasadena , CA 91125 , USA . Email: jlbchamp@caltech.edu

## Abstract

Mass spectrometric glycan rearrangement is problematic because it provides misleading structural information. Here we report on a new reagent, a methylated free radical activated glycan sequencing reagent (Me-FRAGS), which combines a free radical precursor with a methylated pyridine moiety that can be coupled to the reducing terminus of glycans. The collisional activation of Me-FRAGS-derivatized glycans generates a nascent free radical that concurrently induces abundant glycosidic bond and cross-ring cleavage without the need for subsequent activation. The product ions resulting from glycan rearrangement, including internal residue loss and multiple external residue losses, are precluded. Glycan structures can be easily assembled and visualized using a radical driven glycan deconstruction diagram (R-DECON diagram). The presence and location of *N*-acetylated saccharide units and branch sites can be identified from the characteristic dissociation patterns observed only at these locations. The mechanisms of dissociation are investigated and discussed. This Me-FRAGS based mass spectrometric approach creates a new blueprint for glycan structure analysis.

## Abstract 2

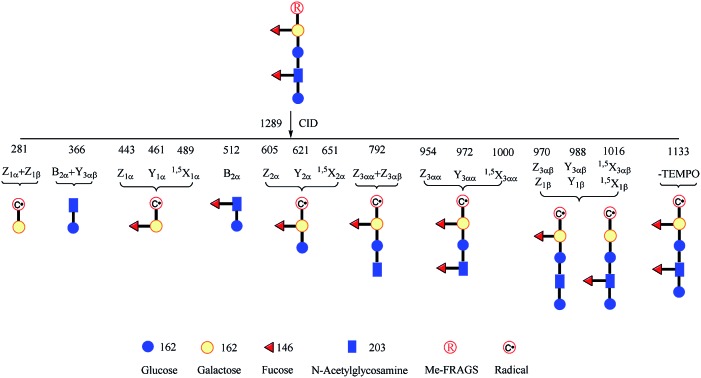
We designed and synthesized a methylated free radical activated glycan sequencing reagent (Me-FRAGS) for eliminating mass spectrometric glycan rearrangement.

## Introduction

Glycosylation is the most common and important post-translational modification of proteins, and plays a central role in biology.^1^ Glycans significantly influence protein folding, activity, stability, solubility, trafficking, localization and oligomerization, and often have intimate involvement in intercellular and intracellular interactions.^2^ Glycans can also be maliciously utilized by tumor cells to evade the immune system, and glycan structural alteration of glycoproteins has been found to occur in various tumors.^3^–^6^ Therefore, elucidating the structure of glycans is essential for the understanding of their functions at a molecular level and thus benefits biomedical research. However, unlike DNA, RNA, and proteins, glycans often exhibit complicated structures with branches and a large number of subunits with both structural and stereochemical diversity. Therefore, glycomics, referring to the systematic study of all the glycan structures of a given cell type or organism, is much less developed than its siblings proteomics and genomics.^7^,^8^

Many techniques including high-performance liquid chromatography (HPLC),^9^–^11^ electrophoresis,^12^,^13^ ion mobility,^14^–^17^ and nuclear magnetic resonance (NMR)^18^,^19^ have been employed for glycan structural analysis. However, these methods have their own limitations. HPLC, electrophoresis, and ion mobility need well-characterized glycan standards. Currently, glycan structure elucidation using these techniques is hampered by the lack of well-characterized glycan standards with structural and stereochemical diversity. NMR requires relatively large quantities of a highly pure sample, and the interpretation of the NMR spectra is difficult due to the similar chemical environments of many protons.

Mass spectrometry has been utilized broadly for glycan structural analysis because the technique requires minimal sample, provides high sensitivity, and enables structural analysis *via* multiple-stage tandem mass spectrometry. Many dissociation techniques, such as collision-induced dissociation (CID),^20^,^21^ infrared multiphoton dissociation (IRMPD),^21^,^22^ higher-energy collisional dissociation (HCD, a specific CID technique),^23^,^24^ ultraviolet multiphoton dissociation,^25^–^27^ electron capture dissociation (ECD),^21^,^28^–^30^ electron transfer dissociation (ETD),^31^,^32^ electron detachment dissociation (EDD),^21^,^31^,^33^,^34^ and electronic excitation dissociation (EED),^35^,^36^ have been demonstrated to provide complementary and extensive information for glycan structural analysis. Electron activated dissociation (ExD) techniques, including ECD, ETD, EDD, and EED, have shown especially great promise for glycan structural characterization.^37^ Free radical chemistry^38^ has also gained great attention in the field of biomolecule analysis using mass spectrometry.^39^ The photolysis of highly labile radical precursors and collisional activation of free radical initiators are the two main methods that have been developed to generate free radical species from the gas-phase ions of biomolecules.^40^–^44^ Recently, these two approaches have been applied to the field of glycan structural characterization and glycan isomer discrimination.^45^,^46^ However, it was found that certain glycans with terminal fucose subunits can undergo rearrangement *via* the migration of the fucose residue, resulting in ambiguous results for glycan structural interpretation.^45^,^46^

Molecular rearrangement is a double-edged sword in the field of mass spectrometry. Predictable rearrangements, such as the McLafferty rearrangement,^47^ yield valuable structural information, while unpredictable rearrangements mislead the structural analysis. Fucose migration, known as “internal residue loss (IRL)”, is the most common rearrangement observed upon the activation of gas-phase glycan ions and is not predictable.^48^–^50^ Fucose, a deoxyhexose lacking a hydroxyl group on the carbon in the C6-position, is one of the most interesting glycan subunits and mostly exists as a terminal modification of oligosaccharides that are not further elongated. Terminal fucosylation is involved in a wide variety of biological and pathological processes, and is one of the most common and important types of glycosylation in cancer and inflammation.^51^–^53^ Alterations in fucosylation levels and patterns have been reported to be linked to tumor progression, offering potential biomarkers for the detection of cancer.^51^–^53^ It is therefore of great importance to avoid mass spectrometric fucose migration to obtain accurate glycan structures. Although different mechanisms have been proposed for fucose migration, the labile proton appears to play a pivotal role in the proposed dissociation pathways.^48^,^50^,^54^

**Scheme 1:**
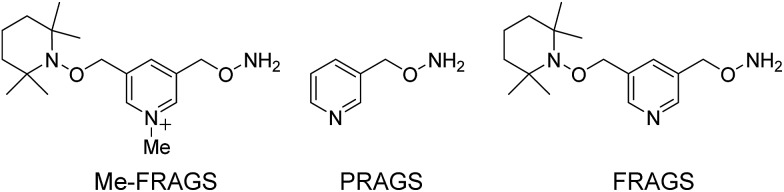
Me-FRAGS, and two previously reported reagents, PRAGS and FRAGS.

To address this problem, we designed and synthesized a methylated free radical activated glycan sequencing reagent (Me-FRAGS, Scheme 1), which contains a free radical precursor and a fixed charge on a pyridine moiety. Like the PRAGS and FRAGS reagents described previously,^45^,^55^ the Me-FRAGS reagent reacts selectively with aldehyde and keto groups and thus targets glycans for regiospecific derivatization at the reducing terminus. Three pairs of glycan isomers, differing only in the location of the fucose subunit in each pair, are employed here to test the capability of the Me-FRAGS reagent.

## Experimental section

### Glycans and reagent

2′-Fucosyllactose (2′-FL), 3-fucosyllactose (3-FL), lacto-*N*-fucopentaose I (LNFP I), lacto-*N*-difucohexaose I (LNDFH I), and lacto-*N*-difucohexaose II (LNDFH II) were purchased from Sigma-Aldrich (St. Louis, MO, USA). Lacto-*N*-fucopentaose V (LNFP V) was purchased from Carbosynth Limited (Berkshire, UK). All solvents are HPLC grade and were purchased from EMD Merck (Gibbstown, NJ, USA). All other chemicals were purchased from Sigma-Aldrich (St. Louis, MO, USA). The synthesis of the Me-FRAGS reagent and the glycan derivatization were achieved according to the previously reported procedures.^45^ The introduction of the methyl group on the pyridine moiety was achieved by allowing FRAGS to react with iodomethane in ether followed by simple ether washing to purify the Me-FRAGS reagent.

### Mass spectrometry

A Thermo-Fisher Scientific linear quadrupole ion trap (LTQ-XL) mass spectrometer (Thermo, San Jose, CA, USA) equipped with an electrospray ionization (ESI) source was employed. Derivatized glycan sample solutions were directly infused to the ESI source of the mass spectrometer *via* a syringe pump at a flow rate of 5 μL min^–1^. The critical parameters of the mass spectrometer include a spray voltage of 5–6 kV, capillary voltage of 30–40 V, capillary temperature of 275 °C, sheath gas (N_2_) flow rate of 10 (arbitrary unit), and tube lens voltage of 50–200 V. Other ion optic parameters were optimized using the auto-tune function in the LTQ-XL tune program for maximizing the signal intensity. CID was performed with resonance excitation of the selected ions for 30 ms. The normalized CID energy was 7–35 (arbitrary unit).

## Results and discussion

All product ions are classified according to the Domon and Costello nomenclature.^56^ The Greek letters α and β are employed to differentiate a branched glycan wherein α indicates the heavier branch and β indicates the lighter branch.

### 2′-Fucosyllactose and 3-fucosyllactose

**Fig. 1:**
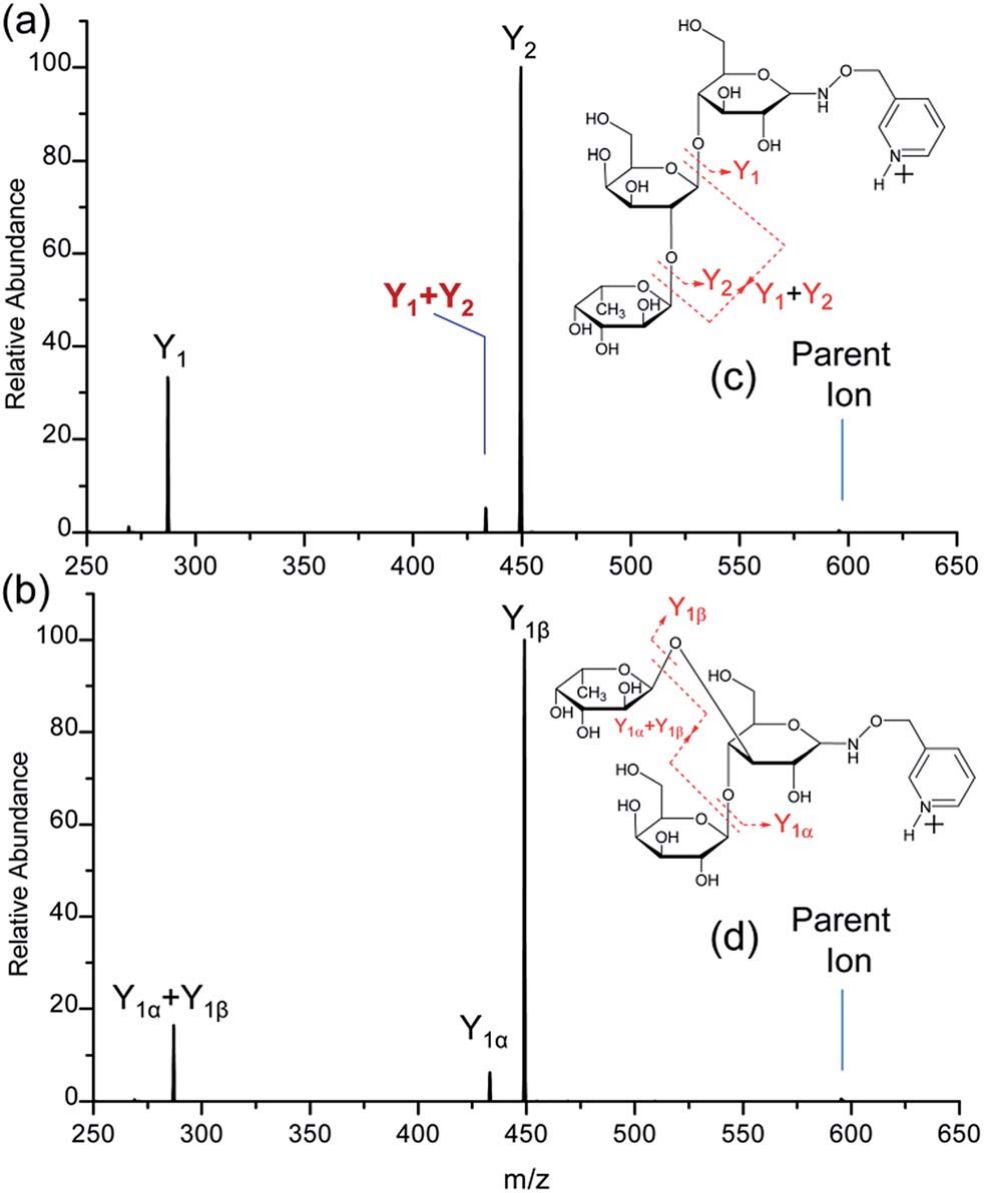
The CID spectra of singly-protonated PRAGS-derivatized 2′-fucosyllactose (a) and 3-fucosyllactose (b), and the fragmentation patterns observed following the CID of singly-protonated PRAGS-derivatized 2′-fucosyllactose (c) and 3-fucosyllactose (d). Parent ion refers to the protonated molecular ion.

2′-Fucosyllactose (2′-FL) is the most prevalent human milk oligosaccharide (HMO).^57^ Interestingly, in cases where 2′-FL is absent, 3-fucosyllactose (3-FL) is present in the highest concentration.^58^ 2′-FL and 3-FL differ only in the location of the fucose subunit. The fucose subunit is bonded to the galactose subunit (non-reducing terminal subunit) of lactose through an α1-2 linkage in 2′-FL while the fucose subunit is bonded to the glucose subunit (reducing terminal subunit) of lactose *via* an α1-3 linkage in 3-FL (Fig. 1).

### CID of PRAGS-derivatized 2′-FL and 3-FL ions

As expected, only Y-type ions, arising from glycosidic bond cleavage, were observed in the CID spectra of PRAGS-derivatized 2′-FL and 3-FL (Fig. 1). Y ions are formed *via* a proton catalyzed mechanism as proposed previously.^45^ The fucose migration product ion Y_1_ + Y_2_ for 2′-FL, representing the internal residue loss (IRL) of galactose, has the same mass as the product ion Y_1α_ for 3-FL. Initially, Ma *et al.* proposed a mechanism to account for the migration of fucose from the C2 position of the central residue of a trisaccharide such as 2′-FL.^54^ This mechanism involves the protonation of the oxygen atoms within the sugar ring, the cleavage of the adjacent C–O bond to give a carbonium ion at the C1 position of the migrating sugar ring, and the subsequent nucleophilic attack of the oxygen atom of the glycosidic bond. Since this mechanism cannot be used to explain the migration from the C3 or C4 position because of the rigidity of the system, Harvey *et al.* introduced another mechanism in which the nitrogen atom of the derivatization reagent instead of the oxygen atom of the glycosidic bond would act as the nucleophile.^48^

In addition to the IRL, the assignment of the glycan structure can be complicated by multiple external residue losses (M-ERL). For example, the M-ERL product ion Y_1α_ + Y_1β_ for 3-FL, which is formed by the external loss of fucose from the lighter chain and the external loss of galactose from the heavy chain simultaneously, has the same mass as the Y_1_ ion for 2′-FL. However, M-ERL draw less attention than internal residue loss (IRL). Here, the Y_1α_ + Y_1β_ ion is proposed to form *via* a cascade Y ion formation mechanism (Scheme S1†).^45^ The IRL and M-ERL jointly contribute to the generation of two similar CID spectra, making the differentiation of these two simple glycan isomers difficult and ambiguous. These rearrangements and sequential losses are also likely to be encountered in the structural analysis of unknown glycans.

### CID of FRAGS-derivatized 2′-FL and 3-FL ions

**Fig. 2:**
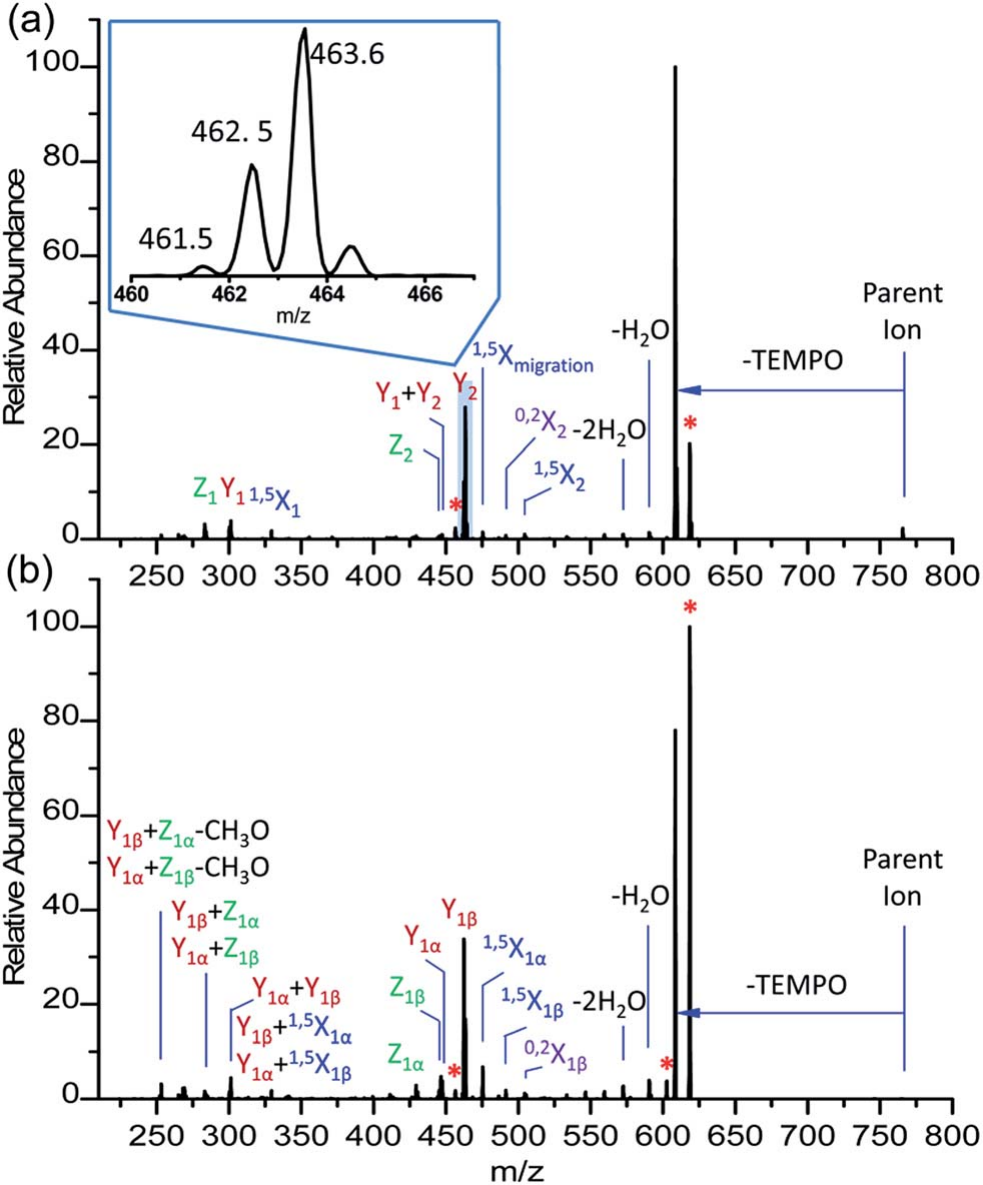
The CID spectra of singly-protonated FRAGS-derivatized 2′-fucosyllactose (a) and 3-fucosyllactose (b). Parent ion refers to the protonated molecular ion. Peaks marked with asterisks are the product ions corresponding to the glycosidic bond cleavage from the parent ion.

**Fig. 3:**
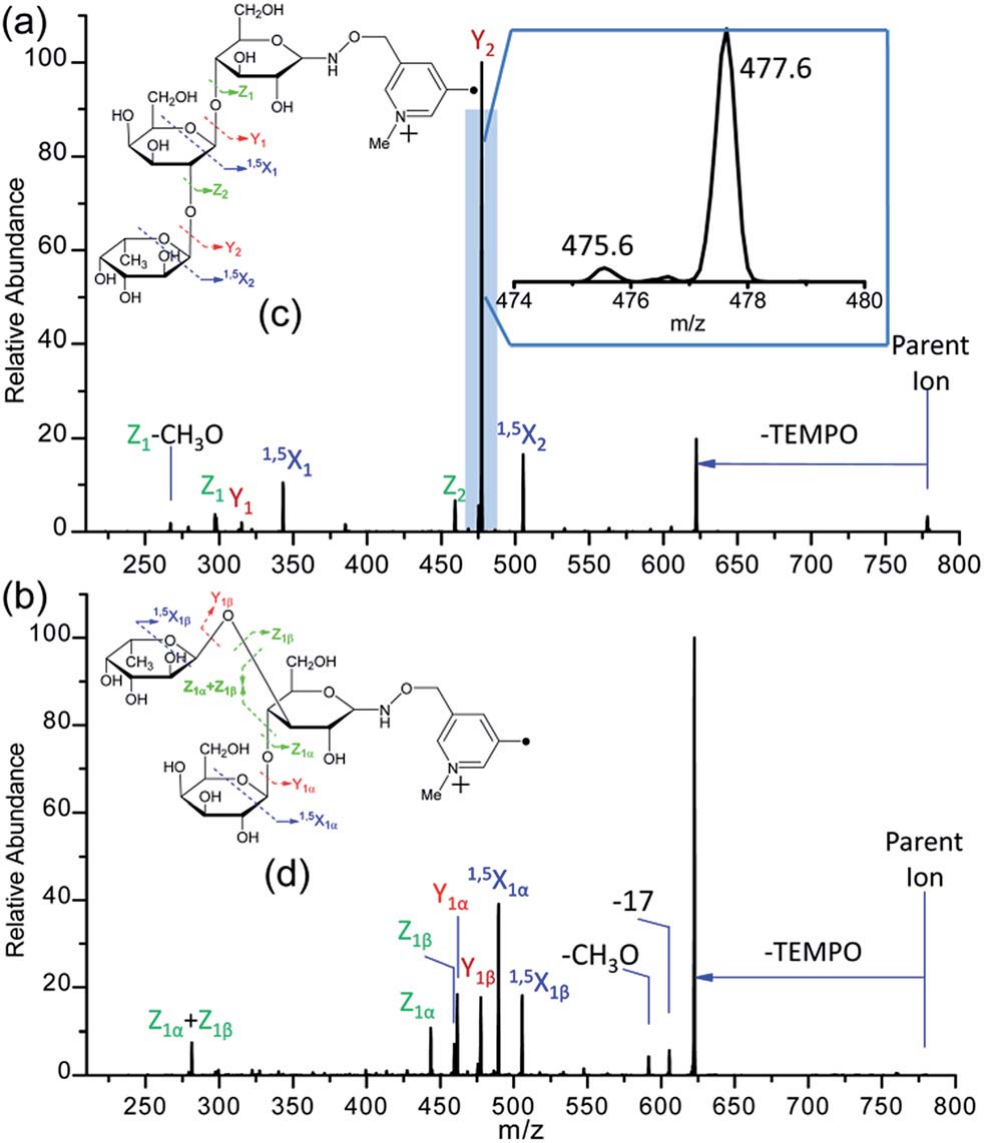
The fragmentation patterns observed following the CID of Me-FRAGS-derivatized 2′-fucosyllactose (c) and 3-fucosyllactose (d), and the CID spectra of Me-FRAGS-derivatized 2′-fucosyllactose (a) and 3-fucosyllactose (b). Parent ion refers to the methylated molecular ion.

The CID spectra of FRAGS-derivatized 2′-FL and 3-FL are more complex than those of their PRAGS-derivatized analogues. More extensive fragmentation, including glycosidic bond cleavage (Y and Z) and cross-ring cleavage (^1,5^X and ^0,2^X), is generated when employing the FRAGS reagent (Fig. 2). The Z, ^1,5^X, and ^0,2^X ions are generated *via* a free radical initiated mechanism.^45^ Meanwhile, as shown in Fig. 2, three types of Y ions (Y_*M*_, Y_*M*+1_, and Y_*M*+2_; subscripts *M* + 1 and *M* + 2 indicate an increase of one and two mass units when cleaving the C1–O glycosidic bond, respectively) are generated upon collisional activation, such as the *m*/*z* 461.5, *m*/*z* 462.5, and *m*/*z* 463.6 ions for Y_2_ cleavage (Fig. 2). The Y_*M*+1_ ion (*m*/*z* 462.5) is formed *via* a proton-catalyzed mechanism as reported previously.^45^ However, the Y_*M*_ and Y_*M*+2_ ions (*m*/*z* 461.5 and 463.6 ions, respectively) are generated *via* a free radical-initiated mechanism. This is confirmed by the formation of the analogous ions, *m*/*z* 475.6 and 477.6 (Fig. 3), when employing the Me-FRAGS reagent. The formation mechanism of these two types of Y ions will be discussed below in the context of the Me-FRAGS reagent. Product ions formed *via* IRL and M-ERL are also observed, such as the Y_1_ + Y_2_ and Y_1α_ + Y_1β_ ions (Fig. 2). Furthermore, the Y* ions, corresponding to the peaks marked with red asterisks in Fig. 2, are generated *via* glycosidic bond cleavage directly from the parent ion with the retention of the radical precursor. These ions increase the complexity of the spectra, making the interpretation of the glycan structure and differentiation of these isomeric glycans ambiguous.

### CID of Me-FRAGS derivatized 2′-FL and 3-FL ions

**Fig. 4:**
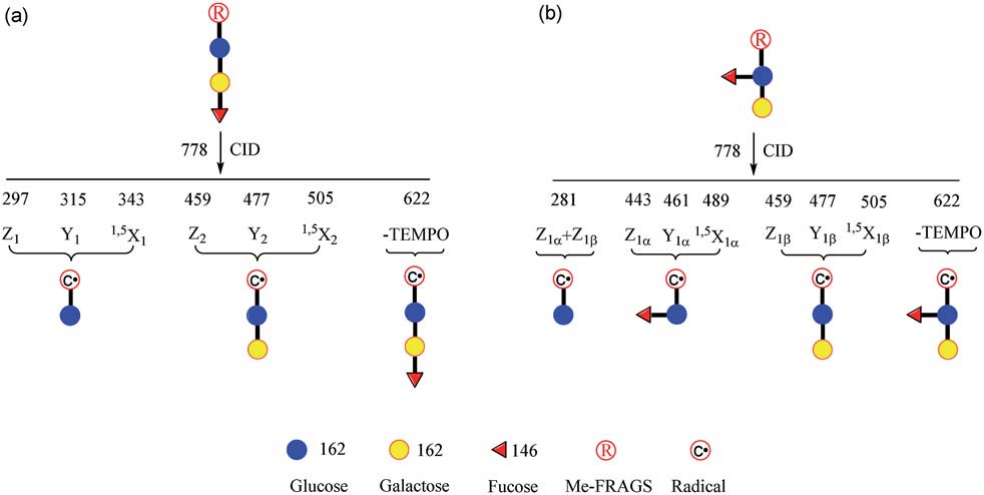
Glycan R-DECON diagrams for Me-FRAGS derivatized 2′-FL (a) and 3-FL (b). The precursor ion (*m*/*z* 778) is subjected to MS^2^ to generate a series of ions with *m*/*z* values from 281 to 622.

It is difficult to distinguish 2′-FL and 3-FL *a priori* through employing the PRAGS and FRAGS reagents because of the fucose migration and sequential external residue losses. Therefore, it is desirable to develop a reagent which can avoid the shortcomings of the PRAGS and FRAGS reagents. As mentioned above, the labile proton is proposed to be the origin of IRL, M-ERL, and Y*. To eliminate the labile proton, the Me-FRAGS reagent was designed and synthesized. As expected, ions generated by IRL and M-ERL and Y* ions are not observed in the CID (MS^2^) spectra (Fig. 3). In addition, the Y + Z and Y + ^1,5^X ions are not formed, significantly decreasing the complexity of the spectra. The formation of the ^0,2^X and ^1,5^X ions is initiated by hydrogen abstraction at different ring positions, and the one that leads to the eventual formation of ^1,5^X is favored.^59^ However, all the essential dissociation patterns (Y- and Z-type glycosidic bond cleavages, ^1,5^X cross-ring cleavages, and Z_α_ + Z_β_ cleavage) are preserved for the determination of the glycan structure. The resulting systematic Me-FRAGS-directed fragmentation of the glycan inspired the development of a radical-driven glycan deconstruction diagram (R-DECON diagram), which visually summarizes the MS^2^ results and thus allows the assembly of the glycan skeleton (Fig. 4).

**Scheme 2:**
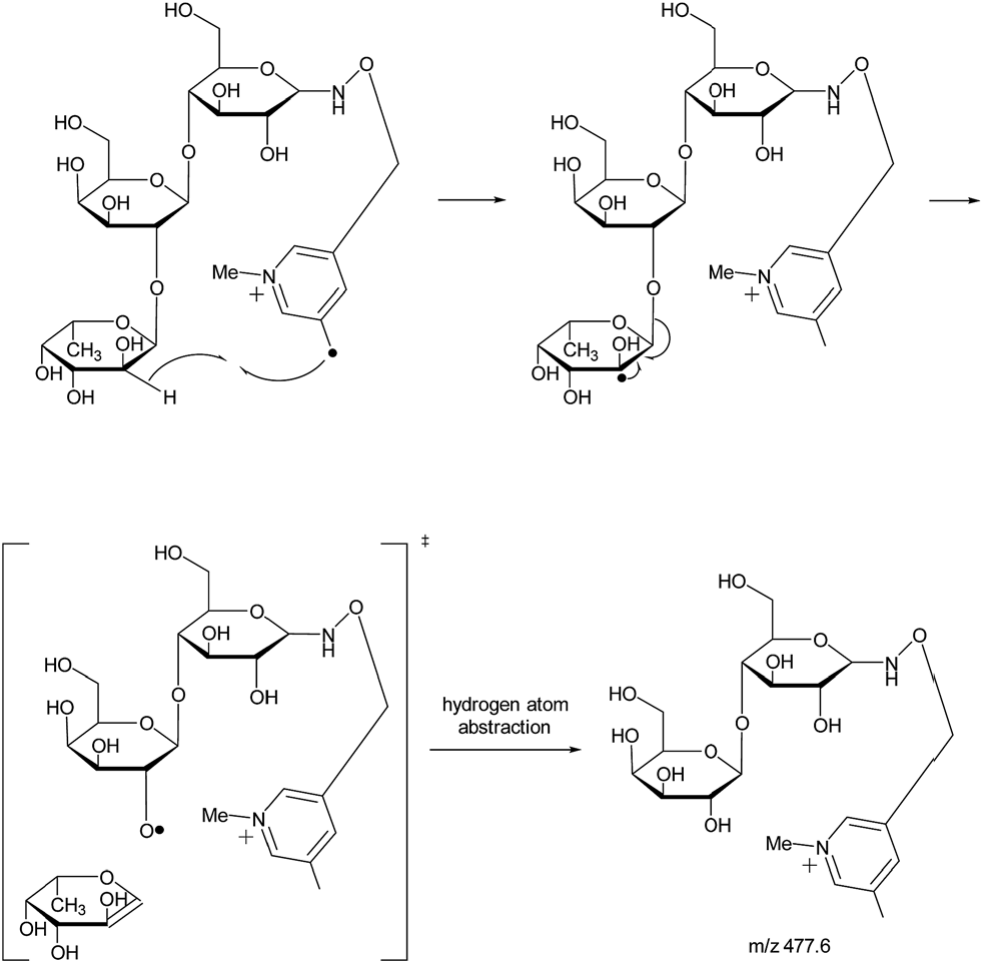
Proposed mechanism for the formation of the Y_*M*+2_ ion.

**Scheme 3:**
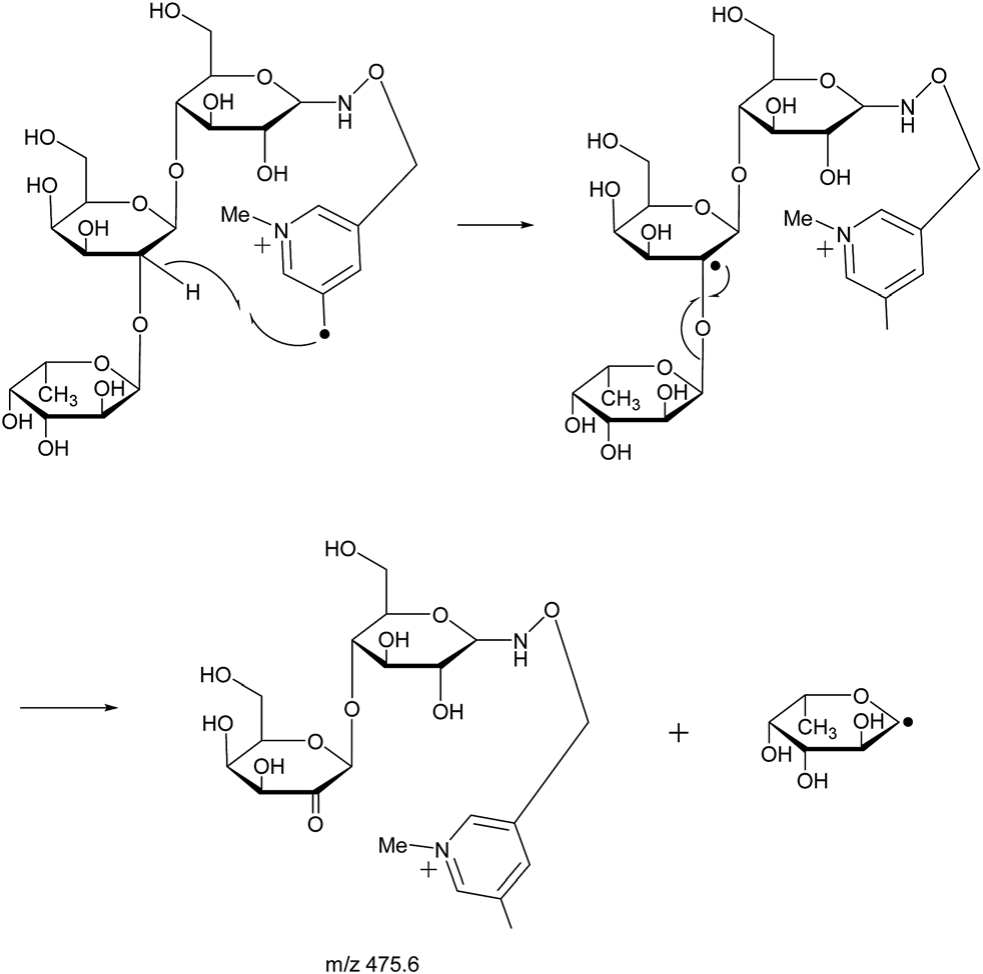
Proposed mechanism for the formation of the Y_*M*_ ion.

With the absence of a labile proton, only two types of Y ions (Y_*M*_ and Y_*M*+2_) are generated *via* a free radical-initiated mechanism (Schemes 2 and 3). The Y_*M*+2_ ion, such as Y_2+2_ (*m*/*z* 477.6, Fig. 3), is generated *via* hydrogen abstraction from the C2 position of the leaving residue followed by β-cleavage and a second hydrogen abstraction from the leaving residue (Scheme 2). The Y_*M*_ ion, such as Y_2_ (*m*/*z* 475.6, Fig. 3), is formed through hydrogen abstraction from the C2 position of the residue that is linked to the leaving residue, followed by β-cleavage (Scheme 3). A radical cascade is an alternative pathway to generate free radicals from glycan residues. It should be noted that the Y_*M*+2_ ion has a much higher abundance than the Y_*M*_ ion for all the C1–O bond cleavages in the CID spectra of Me-FRAGS derivatized 2′-FL and 3-FL. This result is due to the fact that hydrogen abstraction from the C2 position of the residue that is linked to the leaving residue has a higher steric hindrance than hydrogen abstraction from the C2 position of the leaving residue. More importantly, the Z_α_ + Z_β_ ion is observed only at the branched site, providing a diagnostic product ion that identifies the branching structure. As shown in the glycan R-DECON diagrams of Me-FRAGS derivatized 2′-FL and 3-FL, the branch site can also be identified easily by the Y, Z, and ^1,5^X ions (Fig. 4). By preserving the radical chemistry but eliminating the proton-catalyzed chemistry, the Me-FRAGS reagent generates significantly different MS^2^ spectra for 2′-FL and 3-FL, making the differentiation of 2′-FL and 3-FL unambiguous.

### Lacto-*N*-fucopentaose I and V (LNFP I and V), and lacto-*N*-difucohexaose I and II (LNDFH I and II)

**Fig. 5:**
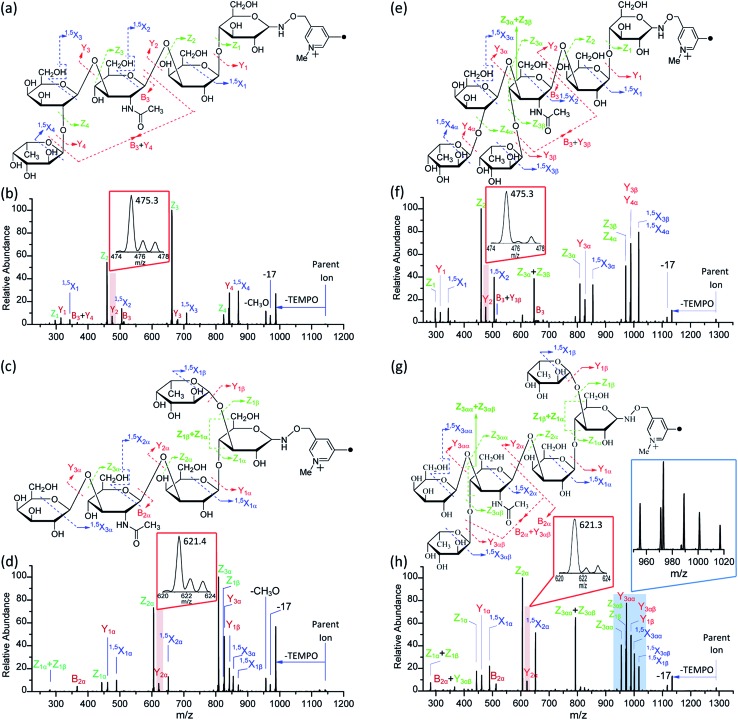
The fragmentation patterns observed following the CID of Me-FRAGS-derivatized LNFP I (a), LNFP V (c), LNDFH I (e), and LNDFH II (g), and the CID spectra of Me-FRAGS-derivatized LNFP I (b), LNFP V (d), LNDFH I (f), and LNDFH II (h). Parent ion refers to the methylated molecular ion.

To further assess the capability of the Me-FRAGS reagent to prevent the structural rearrangement of gas-phase glycan ions and distinguish more complex isobaric glycan structures, the dissociation behavior of two more pairs of HMOs was examined. LNFP I and V are monofucosylated pentasaccharides differing only in the location of the fucose subunit, while LNDFH I and II are difucosylated hexasaccharides differing also only in the location of one fucose subunit (Fig. 5).

### CID of PRAGS derivative ions

As expected, the Y + Y (generated *via* IRL) and Y_α_ + Y_β_ (generated *via* M-ERL) ions are observed following collisional activation (Fig. S1†). It was also found that IRL is not limited to fucose migration; galactose migration (Y_2α_ + Y_3α_ ion for LNFP V and Y_3αα_ + Y_2α_ for LNDFH II) emerged for LNFP V and LNDFH II, both of which contain a terminal galactose residue. Moreover, in addition to single residue migration, the migration of conjunctive glycan subunits was noted, such as the Y_2_ + Y_3_ ion for LNFP I and the Y_3α_ + Y_2_ ion for LNDFH I.^60^ Furthermore, the Y_4α_ + Y_3β_ + Y_2_ ion contains two separate fucose migrations. The IRL observed in these four complex glycans was found to occur regardless of the location of the glycosidic linkage and therefore agrees with the mechanism for glycan internal residue loss proposed by Harvey and co-workers.^48^

### CID of FRAGS derivative ions

Due to the presence of proton-catalyzed and free radical-initiated dissociation, the CID spectra of the FRAGS derivatives are much more complicated than those of their PRAGS-derivatized analogues. More extensive ions, including Y, Z, ^1,5^X, ^0,2^X, Y + Z, Y + ^1,5^X, Y + Y, Y_α_ + Y_β_, Z + Z, B, B + Y, and Y* ions, are generated upon collisional activation (Fig. S2†). The formation of these ions greatly increases the complexity of the spectra and makes the interpretation of the glycan structure challenging.

### CID of Me-FRAGS derivative ions

**Fig. 6:**
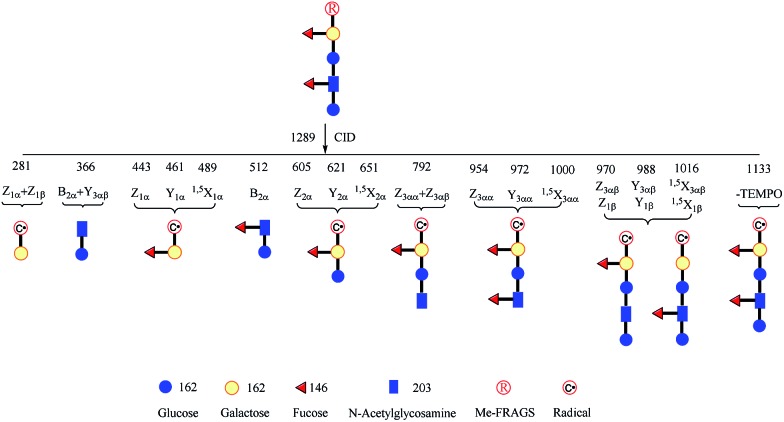
Glycan radical R-DECON diagram for Me-FRAGS derivatized LNDFH II. The precursor ion (*m*/*z* 1289) is subjected to MS^2^ to generate a series of ions with *m*/*z* values from 281 to 1133.

It is difficult to differentiate these two pairs of complex glycan isomers by employing the PRAGS and FRAGS reagents. Fortunately, by employing the Me-FRAGS reagent these two pairs of isobaric glycan isomers can be easily distinguished from the unique fragmentation patterns and R-DECON diagram as described below. Z, Y (Y_*M*_ and Y_*M*+2_), ^1,5^X, and Z_α_ + Z_β_ ions are observed in the CID spectra of the Me-FRAGS-derivatized glycans (Fig. 5). The R-DECON diagram facilitates the straightforward visualization of the glycan skeleton (Fig. 6). Although the intensity of the B and B + Y ions is relatively low, they can be utilized to deduce the presence and locations of *N*-acetylated saccharide residues. Interestingly, as shown in Fig. 5, the Y_*M*_ ion (*m*/*z* 475.3 for LNFP I, *m*/*z* 475.3 for LNDFH I, *m*/*z* 621.4 for LNFP V, and *m*/*z* 621.3 for LNDFH II) is more abundant than the Y_*M*+2_ ion only when the C1_*N*-acetylglucosamine_–O glycosidic bond is cleaved. This can be rationalized by considering the steric hindrance of the *N*-acetyl group at the C2 position of the *N*-acetylglucosamine unit. To some extent, the *N*-acetyl group will block the pathway of hydrogen abstraction from the C2 position of the *N*-acetylglucosamine residue, which is the first step of the mechanism proposed above for the generation of the Y_*M*+2_ ion. This result supports the proposed mechanisms for the formation of the Y_*M*_ and Y_*M*+2_ ions, and therefore can also be utilized to verify the existence and location of *N*-acetylated saccharide residues. Again, the Z_α_ + Z_β_ ions (Z_1α_ + Z_1β_ for LNDFH I, and Z_1α_ + Z_1β_ and Z_3αα_ + Z_3αβ_ for LNDFH II) verify the branch sites in the structure. The relative abundance of Z_α_ + Z_β_ increases greatly through eliminating the acid–base chemistry, which supports the radical driven mechanism for the formation of this ion proposed previously.^45^ The absence of internal loss, multiple external losses, Y*, and sequential dissociation ions (Y + Y and Y + ^1,5^X ions) significantly simplifies the CID spectra, making the differentiation of the isobaric glycan isomers unambiguous.

## Conclusion

The capability of the Me-FRAGS reagent to eliminate glycan rearrangement and thus distinguish isobaric glycans is demonstrated. The PRAGS and FRAGS reagents fail to yield unambiguous structural assignment because of the generation of misleading dissociation products, such as Y + Y and Y_α_ + Y_β_ ions, arising from IRL and M-ERL, respectively. The participation of the labile proton is proposed to account for the IRL and M-ERL. By substituting a fixed charge for the labile proton *via* the methylation of the pyridine moiety of the Me-FRAGS reagent, Y + Y ions (generated by IRL), Y_α_ + Y_β_ ions (generated by M-ERL), Y* ions, and Y + Z and Y + ^1,5^X ions are eliminated, significantly decreasing the complexity of the spectra. Meanwhile, all the essential fragmentation patterns (Y, Z, ^1,5^X, Z_α_ + Z_β_, B, and B + Y) are preserved for the determination of the glycan structure. The incorporation of the observed product ions into an R-DECON diagram provides a simple method to assign and visualize glycan structure. Therefore, isobaric glycans differing in the locations of the terminal glycan residues can be readily distinguished. The branch sites within glycans can be deduced easily either from the characteristic Z_α_ + Z_β_ ion or through the R-DECON diagram. B and B + Y ions can be utilized to infer the presence and location of *N*-acetylated saccharide residues. Y_*M*_ and Y_*M*+2_ are the two types of C1–O glycosidic bond cleavage ions induced by the nascent free radical. The proposed mechanisms for the generation of Y_*M*_ and Y_*M*+2_ are supported by the C1_*N*-acetylglucosamine_–O glycosidic bond cleavage, wherein Y_*M*_ has a higher intensity than Y_*M*+2_. This finding can be used for the identification of *N*-acetylated saccharide residues.

By utilizing the Me-FRAGS reagent, any instrument with the capability of MS^2^ can be employed for glycan structural characterization and isobaric glycan differentiation. The high fragmentation efficiency and systematic radical-directed fragmentation facilitate its application in addressing problems in structural glycobiology.

## Supplementary Material

Supplementary information
