# Supplementary material for: Eradicating mass spectrometric glycan rearrangement by utilizing free radicals
Source: Chem Sci. 2016 May 5;7(8):5390–7. doi: 10.1039/c6sc01371f (PMC6020757; doi:10.1039/c6sc01371f)
Supplement: Supplementary file 1 [file SC-007-C6SC01371F-s001.pdf]

Electronic Supplementary Material (ESI) for Chemical Science.
This journal is © The Royal Society of Chemistry 2016

Eradicate Mass Spectrometric Glycan Rearrangement by Utilizing Free Radical

Nikunj Desai,[a] Daniel A. Thomas,[b] Jungeun Lee,[a] Jinshan Gao,*[a] J. L. Beauchamp*[b]

[a] Department of Chemistry and Biochemistry, and Center for Quantitative Obesity Research, Montclair State University, Montclair, NJ 07043

[b] Arthur Amos Noyes Laboratory of Chemical Physics, California Institute of Technology, Pasadena, CA 91125

*To whom correspondence should be addressed: E-mail: gaoj@montclair.edu, jlbchamp@caltech.edu.

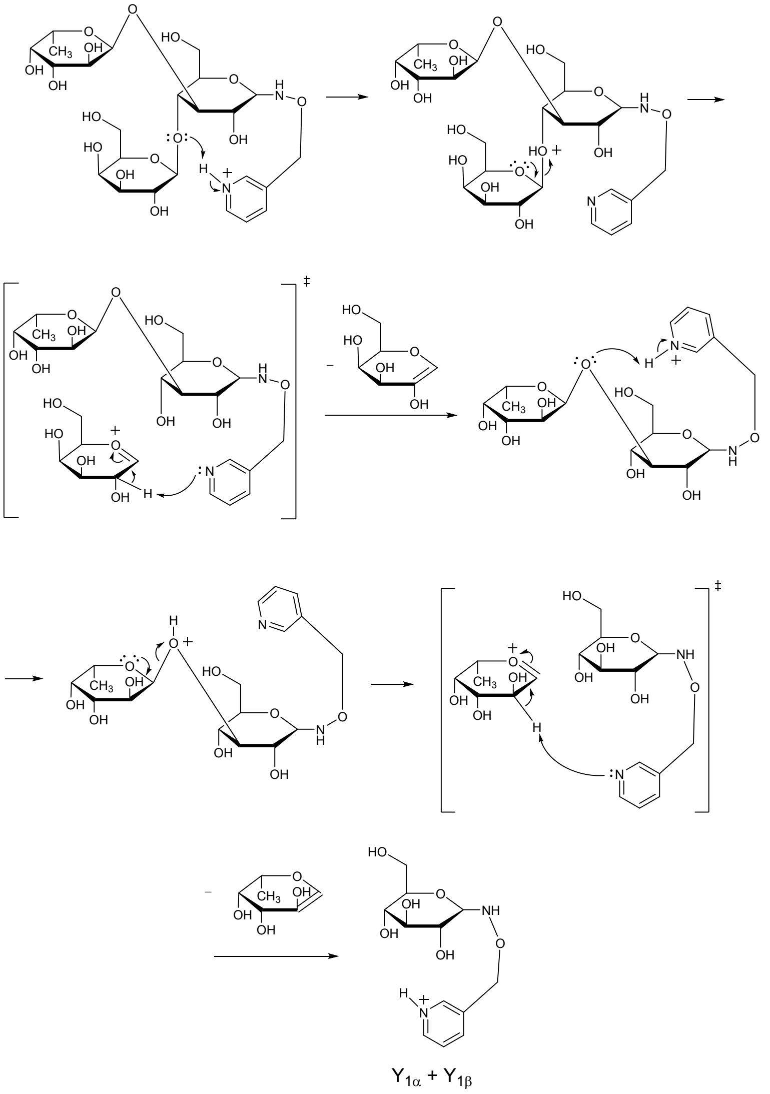

Scheme S1. Proposed mechanism for the formation of $Y_{1\alpha}$+$Y_{1\beta}$ ion.

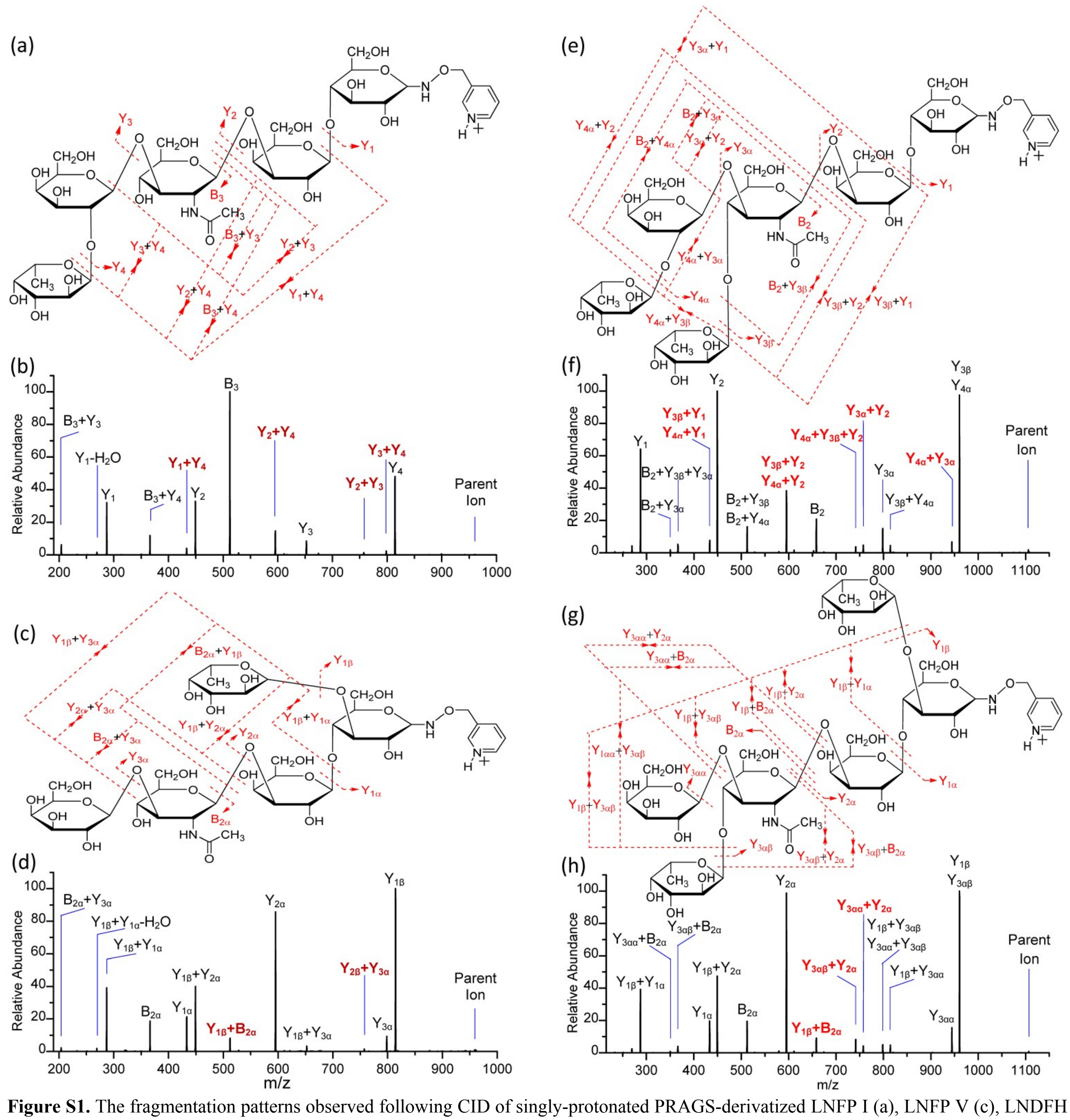

Figure S1. The fragmentation patterns observed following CID of singly-protonated PRAGS-derivatized LNFP I (a), LNFP V (c), LNDFH I (e), and LNDFH II (g), and the CID spectra of singly-protonated PRAGS-derivatized LNFP I (a), LNFP V (c), LNDFH I (e), and LNDFH II (g),. Parent ion refers to the protonated molecular ion.

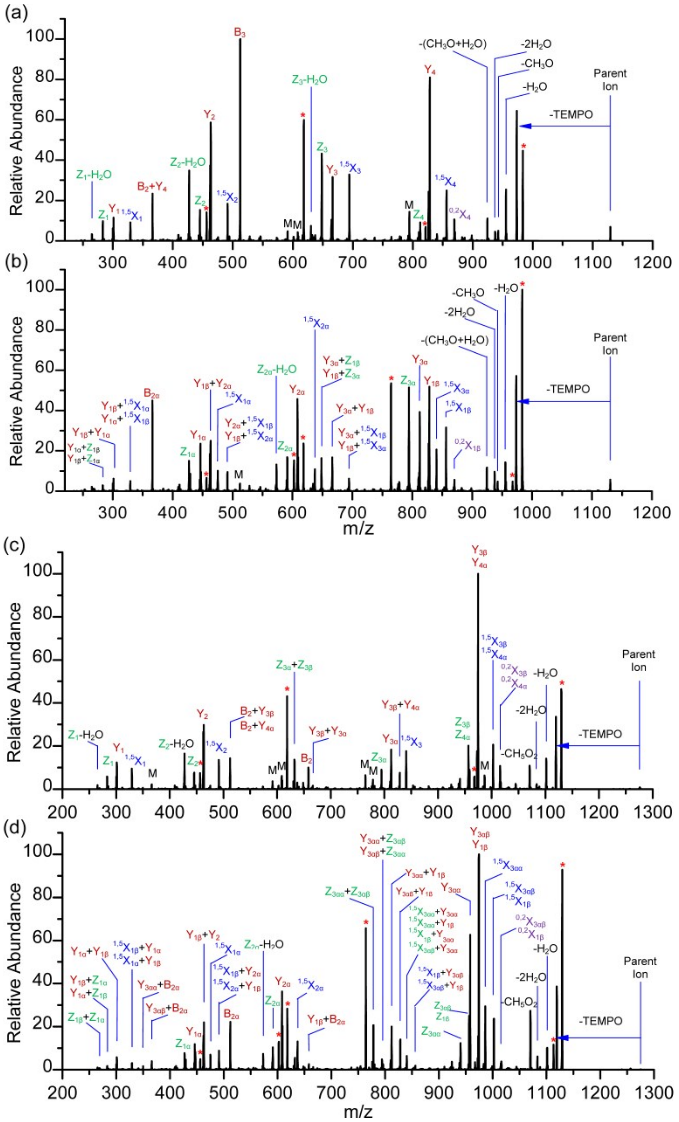

Figure S2. The CID spectra of singly-protonated FRAGS-derivatized LNDFH I (a), LNDFH II (b), LNFP I (c), and LNFP V (d). Parent ion refers to the protonated molecular ion.
